# The Associations between Toll-Like Receptor 9 Gene Polymorphisms and Cervical Cancer Susceptibility

**DOI:** 10.1155/2018/9127146

**Published:** 2018-07-25

**Authors:** Sijuan Tian, Liping Zhang, Ting Yang, Xing Wei, Li Zhang, Yang Yu, Yang Li, Di Cao, Xiaofeng Yang

**Affiliations:** ^1^Department of Obstetrics and Gynecology, The First Affiliated Hospital of Xi'an Jiaotong University, 277 West Yanta Road, Xi'an, Shaanxi 710061, China; ^2^Department of Obstetrics and Gynecology, Taihe Hospital, Hubei University of Medicine, Shiyan, Hubei 442000, China

## Abstract

This meta-analysis systematically reviews the association between Toll-like receptor 9 polymorphisms and the risk of cervical cancer. Case-control studies focused on the association were collected from the PubMed, Web of Science, Cochrane Library, Embase, MEDLINE, CNKI, VIP, and Wanfang databases from inception to July 2017. We screened the studies and assessed the methodological quality of the included studies and extracted data. A meta-analysis was performed using RevMan 5.3 and Stata 12.0 software. Pooled odds ratios and 95% confidence intervals were employed to evaluate the strength of the associations between Toll-like receptor 9 polymorphisms and cervical cancer risk. A total of 9 studies comprising 3331 cervical cancer patients and 4109 healthy controls met the inclusion criteria. Of these, 8 studies contained information about G2848A (rs352140) and 4 studies contained information about −1486T/C (rs187084). Our results revealed that the associations between rs187084 and cervical cancer risk in the dominant model (*p* = 0.002) and heterozygous model (*p* = 0.002) were significant, with 1.30- and 1.32-fold increases in susceptibility, respectively, compared to that in the wild-type model. However, rs352140 was not related to cervical cancer regardless of whether the subgroup analysis was conducted (*p* > 0.05). In conclusion, there is a significant correlation between rs187084 and cervical cancer risk with the minor C allele increasing the risk of occurrence of cervical cancer. However, rs352140 is not associated with the occurrence of cervical cancer.

## 1. Introduction

Cervical cancer is the fourth most common cancer in women in terms of both incidence and mortality worldwide [1]. According to global cancer statistics, there were approximately 527,600 new cervical cancer cases and 265,700 deaths in 2012. In low-income countries, cervical cancer ranks as second in incidence and is the third leading cause of cancer-associated death among women. In China, the estimated new cancer cases and deaths were 98,900 and 30,500, respectively, in 2015, presenting an upward trend [2, 3]. Human papillomavirus (HPV) was identified as a principal cause of cervical cancer [4, 5]. However, 90% of HPV genital infections can be spontaneously cleared, and few of these progress to cervical cancer [6], suggesting that other pathogeneses and aetiologies might contribute to cervical carcinogenesis.

Toll-like receptors (TLRs) are transmembrane proteins that recognize pathogen-associated molecular patterns (PAMPs), conserved structural motifs in bacteria, fungi, and viruses [7]. TLRs initiate the innate immune response and further modulate acquired immune response, playing an important role in inflammation and carcinogenesis. TLR9 recognizes nonmethylated CpG islands in viral DNA and activates the immune system. In the past few years, evidence suggests that *TLR9* expression increases according to the histopathological grade of the cervical pathological process and the HPV E6 and E7 oncoproteins deregulate the expression and function of TLR9 [8, 9].

It has been noted that the *TLR9* gene is polymorphic and associated with various cancers, including cervical cancer, prostate cancer, oesophageal cancer, gastric cancer, breast cancer, colorectal carcinoma, and lymphoma [10]. Many single-nucleotide polymorphisms (SNPs) of *TLR9* have been studied, including rs352140, rs187084, rs352149, rs445676, and rs5743836, according to the NCBI database (https://www.ncbi.nlm.nih.gov/snp). Among these SNPs, two prominent variants, namely, rs352140 (G2848A) and rs187084 (−1486T/C), are frequently observed to be related to cervical cancer susceptibility. However, the results are contentious. Therefore, we conducted this meta-analysis to estimate the association between cervical cancer risk and the most concerning two SNPs of *TLR9*, rs352140 and rs187084.

## 2. Materials and Methods

### 2.1. Literature Search Strategy

We reviewed the PubMed, Web of Science, the Cochrane Library, Embase, MEDLINE, CNKI, VIP, and Wanfang databases systematically and comprehensively. The search terms are the following: Toll-Like Receptors [Mesh] or TLR^∗^, polymorphism^∗^/variant^∗^/mutation^∗^/SNP, Uterine Cervical Neoplasm [Mesh]/cervix cancer/cervical cancer, and the combinations of these. Additionally, we searched the reference lists of all identified articles manually for more studies.

### 2.2. Inclusion and Exclusion Criteria

Studies included needed to meet the following criteria: (1) a focus on the association between the *TLR9* gene polymorphisms (rs352140 and rs187084) and the risk of cervical cancer, (2) human study subjects, (3) case-control studies, (4) available and sufficient genotype distribution of data to calculate odds ratios (ORs) and corresponding 95% confidence intervals (CIs), and (5) diagnoses based on cervical biopsy pathology. Additionally, if there were duplicate studies, we made sure that the most recent or the most complete one was included. If it did not satisfy the criteria above, the article was excluded.

### 2.3. Data Extraction and Synthesis

Two investigators extracted relevant data from all the eligible studies independently. A third reviewer was invited to participate in the work when some disagreement occurred; consensus was ultimately reached by discussion. These characteristics were collected from each study: the first author, publication year, race, total numbers of cases and controls, study design, source of controls, genotyping method, and evidence of HWE in controls.

### 2.4. Quality Assessment

The quality of included studies was assessed using the Newcastle-Ottawa scale, including three categories: selection, comparability, and outcome. Additional eight items were used to assess the methodology of each qualified study. The highest score was 9. Studies with a score of more than 7 were considered as high quality. A study awarded a score of 0–3, 4–6, or 7–9 was considered as a low-, moderate-, or high-quality study, respectively.

### 2.5. Statistical Analysis

The odds ratios (ORs) and 95% confidence interval (CIs) were applied to assess the strength of the correlation between SNPs and cervical cancer susceptibility. A *Z*-test revealed statistical significance when *p* < 0.05. *I*^2^ and *Q* statistics were employed to detect heterogeneity among different studies. There was no heterogeneity if *I*^2^ < 50% and *p* > 0.1 and a fixed effects model was used; otherwise, we thought that heterogeneity existed in the incorporated populations and a random effects model was used instead. Subsequently, we conducted a subgroup analysis according to race. Hardy-Weinberg equilibrium (HWE) was evaluated by *χ*^2^ test in control groups with *p* < 0.05 indicating a deviation from HWE. Sensitivity analysis was utilized to estimate the robustness and stability of the meta-analysis results by deleting all the studies one by one. Next, Begg's funnel plot and Egger's test were used to evaluate publication bias. For each SNP, five genetic models were evaluated to assess the correlation with cervical cancer susceptibility: the allele model, dominant model, recessive model, heterozygote model, and homozygous model. The statistical analyses were performed using RevMan 5.3 and Stata 12.0 software. All *p* values were two sided, and *p* < 0.05 was considered to be statistically significant.

## 3. Results

### 3.1. Characteristics of Included Studies

**Figure 1:**
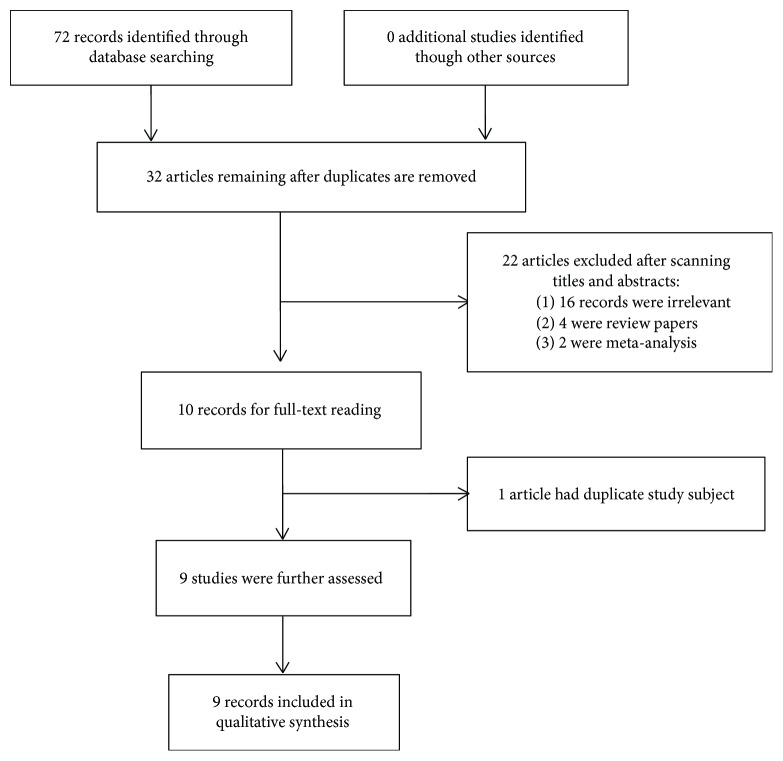
Flow diagram of searching procedure.

By searching the electronic databases systematically, we initially retrieved 72 articles (Figure 1). After excluding duplicate studies, 32 articles remained. Further reviewing of the titles and abstracts of the identified studies allowed the removal of 22 articles. Of those removed, 16 were clearly irrelevant to *TLR9* polymorphisms, 4 were review papers, and 2 were meta-analyses. We downloaded the remaining 10 articles as full-text reports and reviewed them carefully. One record was excluded for containing duplicate samples. Finally, 9 case-control studies containing 3331 cases and 4109 controls were included in the meta-analysis, among which 8 studies were about rs352140 (G2848A) and 4 articles were about rs187084 (−1486T/C). All studies were based on Caucasian or Chinese Han populations.

These 9 case-control studies were published between 2011 and 2017. Four studies were performed with Caucasians, while five papers were based on Chinese Han populations. All the studies were performed using PCR-RFLP to identify the polymorphism sites except two studies, which used the TaqMan and Illumina GoldenGate methods. Two of the nine control groups were hospital-based groups, while the rest were population-based groups (Table 1).

We applied the Newcastle-Ottawa scale (NOS) to estimate the quality of the nine included studies [20]. The results showed that all studies were of high quality (Table 1). The distributions of the genotypes and allele frequencies of rs352140 (G2848A) and rs187084 (−1486T/C) are shown in Table 2. The distributions of the genotypes in the nine control groups were in accordance with Hardy-Weinberg equilibrium (HWE) except for two studies [15, 16] (Table 2).

### 3.2. Meta-Analysis Results

**Figure 2:**
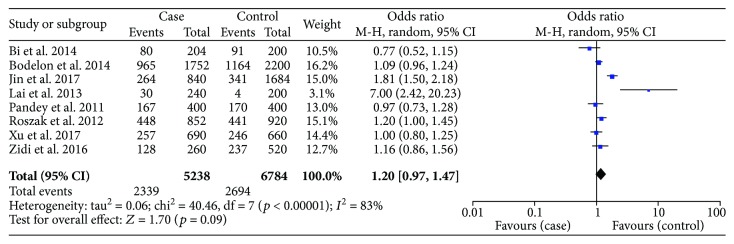
Forest plots of the association between *TLR9* rs352140 polymorphism and cervical cancer risk in the allele genetic model.

**Figure 3:**
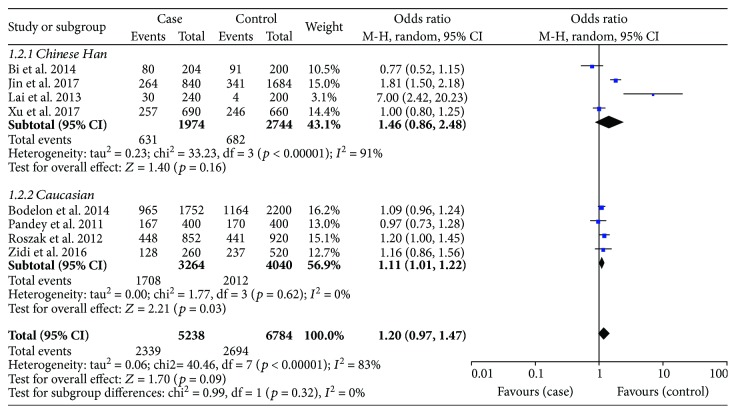
Subgroup analysis of the association between *TLR9* rs352140 polymorphism and the risk of cervical cancer stratified by race in the allele genetic model.

There were 8 studies on rs352140 that included 2619 cases and 3392 healthy controls. The meta-analysis results did not show a statistical relationship between rs352140 and the risk of cervical cancer in any of the five genetic models: allele model (OR = 1.20, *p* = 0.09) (Figure 2), dominant model (OR = 1.30, *p* = 0.08), recessive model (OR = 1.23, *p* = 0.34), heterozygote model (OR = 1.24, *p* = 0.10), or homozygous genetic model (OR = 1.34, *p* = 0.23) (Table 3). Considering the heterogeneity among studies, a subgroup analysis was performed and stratified by race. As presented in Table 3, the results were still stable (*p* > 0.05), but the A allele was correlated to increasing cervical cancer susceptibility in Caucasians based on the allele genetic model (OR = 1.11, *p* = 0.03) (Figure 3). After excluding the two studies that were not in accordance with HWE, the pooled results did not change (data not shown).

**Figure 4:**
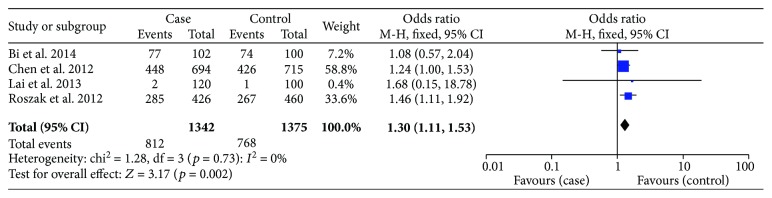
Forest plots of the association between *TLR9* rs187084 polymorphism and cervical cancer risk in the dominant genetic model.

**Figure 5:**
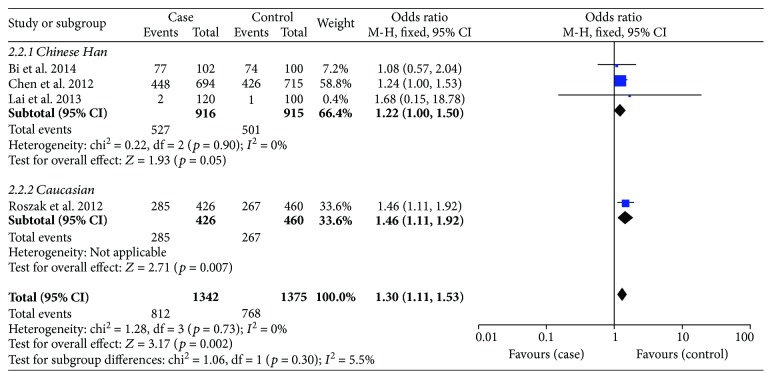
Subgroup analysis of the association between *TLR9* rs187084 polymorphism and cervical cancer risk stratified by race in the dominant genetic model.

**Figure 6:**
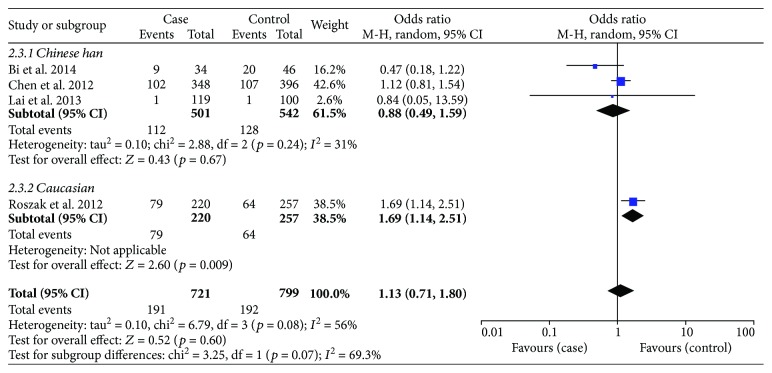
Subgroup analysis of the association between *TLR9* rs187084 polymorphism and cervical cancer risk stratified by race in the homozygous genetic model.

With regard to rs187084, there were 4 records involving 1342 cases and 1375 controls. The pooled ORs suggested a significant association between the SNP and cervical cancer risk: allele model (OR = 1.15, *p* = 0.02), dominant model (OR = 1.30, *p* = 0.002) (Figure 4), and heterozygote model (OR = 1.32, *p* = 0.002); nevertheless, it was not related to cancer susceptibility on the recessive model or homozygous genetic model (*p* > 0.05) (Table 4). We then performed subgroup analysis based on ethnicity to reassess the relationship between rs187084 and cervical cancer risk. The results were stable. Data revealed that *TLR9* rs187084 was related to cervical cancer risk of Chinese Han population in the dominant model (OR = 1.22, *p* = 0.05) (Figure 5) and heterozygous genetic model (OR = 1.28, *p* = 0.02). There was no statistical correlation in the recessive model (OR = 0.70, *p* = 0.34) or homozygous genetic model (OR = 0.88, *p* = 0.67) (Figure 6, Table 4). Furthermore, the same results were present after eliminating a study that deviated from HWE.

With regard to rs187084, there were 4 records involving 1342 cases and 1375 controls. The pooled ORs suggested a significant association between the SNP and cervical cancer risk: allele model (OR = 1.15, *p* = 0.02), dominant model (OR = 1.30, *p* = 0.002) (Figure 4), and heterozygote model (OR = 1.32, *p* = 0.002). However, the SNP was not related to cancer susceptibility in the recessive model or homozygous genetic model (*p* > 0.05) (Table 4). We performed a subgroup analysis based on race to reassess the relationship between rs187084 and cervical cancer risk; the results were stable. Data revealed that *TLR9* rs187084 was related to cervical cancer risk in the Chinese Han population in the dominant model (OR = 1.22, *p* = 0.05) (Figure 5) and heterozygous genetic model (OR = 1.28, *p* = 0.02). There was no statistical correlation in the recessive model (OR = 0.70, *p* = 0.34) or homozygous genetic model (OR = 0.88, *p* = 0.67) (Figure 6, Table 4). Furthermore, the same results were present after eliminating a study that deviated from HWE.

### 3.3. Detection for Heterogeneity

As presented in Table 3, there was great heterogeneity among studies relating to rs352140 in all genetic models (*I*^2^ > 50%, *p* < 0.1). In consideration of this, we employed a random effects model for the meta-analysis. Additionally, the subgroup analysis was stratified by race to eliminate heterogeneity (Figure 3). It was clearly decreased in Caucasians as shown: allele model (*I*^2^ = 0%, *p* = 0.62), dominant model (*I*^2^ = 17%, *p* = 0.30), recessive model (*I*^2^ = 0%, *p* = 0.39), heterozygote model (*I*^2^ = 38%, *p* = 0.20), and homozygous genetic model (*I*^2^ = 0%, *p* = 0.42). This implied that race might be a confounding factor and source of heterogeneity, whereas the pooled ORs were robust substantially.

As for heterogeneity among studies on rs187084, there was no significant heterogeneity in the allele model (*I*^2^ = 43%, *p* = 0.15), dominant model (*I*^2^ = 0%, *p* = 0.73), or heterozygous genetic model (*I*^2^ = 0%, *p* = 0.95). However, heterogeneity was present in the recessive model (*I*^2^ = 63%, *p* = 0.04) and homozygous genetic model (*I*^2^ = 56%, *p* = 0.08). Similarly, a random effects model and subgroup analysis were conducted to evaluate heterogeneity. We found that the heterogeneity decreased in the Chinese Han population based on the homozygous genetic model (*I*^2^ = 31%, *p* = 0.24) (Figure 6), but no significant differences were evident in the pooled results (Table 4).

### 3.4. Sensitivity Analysis

**Figure 7:**
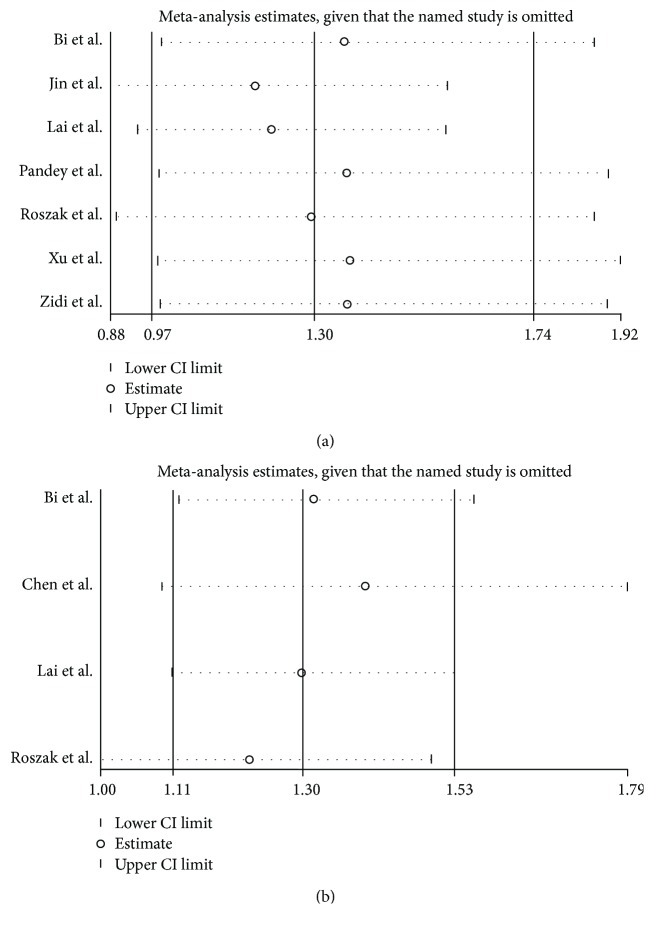
Sensitivity analysis of the association between *TLR9* SNPs and risk of cervical cancer in the dominant genetic model. (a) rs352140. (b) rs187084.

As mentioned before, two studies focused on rs352140 and one focused on rs187084 were not consistent with the balance of HWE in the control groups (*p* < 0.05). However, the results did not change substantially after removing them. Furthermore, a sensitivity analysis was utilized to evaluate the stability of the meta-analysis by deleting all the studies one by one. However, the overall ORs did not change significantly in any of the genetic models, indicating that the meta-analysis was robust and stable. The sensitivity analysis of the associations between the two SNPs and cervical cancer susceptibility based on the dominant genetic model is shown in Figures 7(a) and 7(b).

### 3.5. Publication Bias

**Figure 8:**
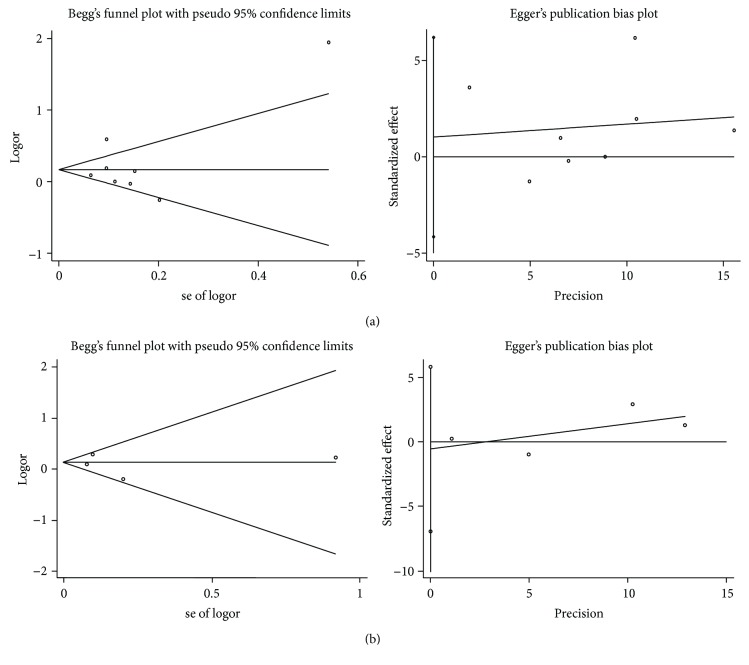
Publication bias of *TLR9* polymorphisms in the allele model. (a) rs352140 (Begg's test: *p* = 1.000, Egger's test: *p* = 0.647). (b) rs187084 (Begg's test: *p* = 1.000, Egger's test: *p* = 0.736).

To detect publication bias, Begg's test and Egger's test were conducted. The results showed that there was no significant evidence of publication bias for either rs352140 or rs187084 based on the five genetic models (*p* > 0.05) (Figures 8(a) and 8(b)).

## 4. Discussion

Persistent infection with HR-HPV is a causative factor in the progression of cervical cancer [21–23]. Cigarette smoking, age, and sexual activity may influence susceptibility to cervical cancer [3, 24].

TLR9 recognizes unmethylated CpG DNA and promotes the production of inflammatory cytokines that contribute to tumour immunity [25]. In addition, elevated TLR9 levels with persistent HPV infection can drive inflammation, thereby contributing to cervical cancer risk [26]. In recent years, a number of studies have focused on *TLR9* gene polymorphisms and cervical cancer susceptibility, but the conclusions are questionable. For rs352140, several studies revealed that rs352140 was not related to cervical cancer; however, the study of Lai et al. supported that rs352140 was associated with an increased risk of cervical cancer, and the study of Pandy et al. showed that A allele had borderline significance conferring a marginally increased risk for advanced cervical cancer [11, 15, 16, 18]. With regard to rs187084, some studies indicated that rs352140 contributed to cervical cancer susceptibility, while the study of Bi et al. showed that the SNP was not associated with cervical carcinogenesis [11, 13]. The findings were even controversial when focused on the same ethnic group. Xu et al. and Bi et al. agreed that there was no relationship between rs352140 and the risk of cervical cancer, while Lai et al. and Jin et al. held the opposite point of view [11, 14, 15, 18]. Similarly, the studies of Lai et al. and Bi et al. indicated that rs187084 was not related to cervical cancer, while the study of Chen et al. supported a different conclusion [11, 13, 15].

Our results indicated that rs352140 is not associated with cervical cancer risk, whereas rs187084 increases susceptibility to cervical cancer. As shown in Table 4, rs187084 is associated with cervical cancer susceptibility in the allele model, dominant genetic model, and heterozygous genetic model but not in the recessive genetic model or homozygous genetic model. We postulated that the minor C allele at the −1486 locus might be a dominant mutation and that C allele carriers had an increased risk for cervical cancer. This is consistent with our conclusion that rs187084 is a risk factor for cervical cancer. However, too many reports of associations between genetic variants and common cancer sites are false positives [27]. Therefore, assessing the false positive report probability (FPRP) and using it to decide whether our finding is deserving of attention are important. Considering that our meta-analysis is a large sample and multicentre study, we applied 0.2 as the cut-off value for FPRP. According to Wacholder's method, we evaluated the FPRPs for the dominant model (CC + CT versus TT) and heterozygous genetic model (CT versus TT). The FPRPs were 0.142 and 0.154, respectively [27]. Obviously, both FPRP results were below the cut-off value, suggesting that rs187084 is related to cervical cancer susceptibility and that our conclusion is noteworthy.

Subgroup analysis of rs352140 showed a significant decline in heterogeneity in the Caucasian population, implying that the heterogeneity may result from racial differences. Moreover, it was statistically significant that the A allele elevated the cervical cancer risk in the allele genetic model (OR = 1.11, *p* = 0.03) while the pooled results in other genetic models remained steady. The evaluated FPRP was 0.751, which was greater than 0.2, implying a false positive association between rs352140 and cervical cancer risk among the Caucasian population [27]. Subgroup analysis and sensitivity analysis of rs187084 were also conducted. The pooled results remained identical, presenting an obvious association between rs187084 and increased cervical cancer risk and suggesting that our meta-analysis results were reliable and stable.

We searched for *TLR9* SNPs in the NCBI database and found that rs352140 was a synonymous codon. This, to some extent, explained the reason for the lack of relevance regarding cervical cancer risk. It has been identified that regulatory polymorphisms controlling gene expression can be localized hundreds of kb away from the genes they influence [28, 29]. In this way, rs187084 is potentially a functional variant located in the *TLR9* promoter region; so, we propose that this SNP contributes to the regulation of *TLR9* at a basal transcript level [30, 31]. Moreover, rs187084 is located near the region that interacts with the HPV16 E6 and E7 oncoproteins [32]. Therefore, it may regulate transcription during HR-HPV infection and increase the risk of cervical cancer. TLR9 plays a fundamental role in pathogen recognition and the activation of innate immunity and was found to be overexpressed in cervical cancer [33, 34]. IFN-*γ* and TNF-*α* are two important cytokines that participate in the innate immune response. In addition to this, they stimulate antigen-presenting cells to express MHC II, enhancing antigen presentation and improving the adaptive immune response. The minor C allele of rs187084 led to significantly lower expression of protective cytokines IFN-*γ* and TNF-*α* than the T allele [35]. In addition, rs187084 C allele increased the transcriptional activity of *TLR9* and provoked a higher level of gene expression [35, 36].

A previous meta-analysis on the relationship between *TLR9* polymorphisms rs352140 and rs187084 and cervical cancer risk conducted by Mu et al. is consistent with our results [37]. There were five studies of rs352140 and four case-control studies of rs187084 in the study of Mu et al. that assessed the correlation between *TLR9* SNPs and cervical cancer risk. Compared to the study of Mu et al., our meta-analysis included three additional qualified studies [14, 18, 19] to assess the association and therefore obtained more reliable and persuasive conclusions. Moreover, in order to reduce the heterogeneity caused by racial differences, we performed a subgroup analysis stratified by race. Thus, our findings are more convincing and rigorous.

Several limitations are present in our meta-analysis. First, even though we retrieved records from electronic databases systematically, there were likely still some unpublished studies we could not acquire because of their negative results, leading to potential bias. Second, the incidence of cervical cancer is highest in sub-Saharan Africa, Latin America, the Caribbean, and Melanesia, where people of African origin account for the majority [1]. However, there were no statistics and studies of interest focused on men of African descent even though several databases were searched. This led to our study focusing on Caucasian and Chinese Han populations, resulting in a bias in the relationship that we are concerned. Additionally, although we considered the effect of HPV on our conclusions and attempted to perform a subgroup analysis, few of the included studies contained HPV data. Therefore, we were unable to conduct a subgroup analysis on HPV infection. Future studies containing more comprehensive information are needed to obtain more reliable conclusions.

In conclusion, this meta-analysis demonstrated that there is no association between *TLR9* rs352140 (G2848A) and cervical cancer susceptibility, while rs187084 (−1486T/C) has a statistical correlation with the risk of cervical cancer. The rs187084 C allele is considered a deleterious allele and increases cervical cancer risk. Further large, well-designed case-control studies are needed to authenticate these results.

## Figures and Tables

**Table 1:** Characteristics of the included studies.

First author	Year	Race	Number (case/control)	Study design	Source of controls	Genotyping method	Study quality (NOS)
Bi [11]	2014	Chinese Han	102/100	CC	Population	PCR-RFLP	7
Bodelon [12]	2014	Caucasian	876/1100	CC	Population	Illumina GoldenGate	8
Chen [13]	2012	Chinese Han	712/717	CC	Population	PCR-RFLP	7
Jin [14]	2017	Chinese Han	420/842	CC	Hospital	PCR-RFLP	7
Lai [15]	2013	Chinese Han	120/100	CC	Hospital	PCR-RFLP	8
Pandey [16]	2011	Caucasian	200/200	CC	Population	PCR-RFLP	7
Roszak [17]	2012	Caucasian	426/460	CC	Population	PCR-RFLP	7
Xu [18]	2017	Chinese Han	345/330	CC	Population	TaqMan	7
Zidi [19]	2016	Caucasian	130/260	CC	Population	PCR-RFLP	8

CC: case-control; PCR: polymerase chain reaction; RFLP: restriction fragment length polymorphism.

**Table 2:** *TLR9* polymorphism genotype distribution and allele frequency in cases and controls.

First author	Genotype (*N*)								Allele frequency (*N*)				HWE (*p* value)
	Case				Control				Case		Control		
2848G>A	Total	GG	GA	AA	Total	GG	GA	AA	G	A	G	A
Bi	102	33	58	11	100	31	47	22	124	80	109	91	0.601
Bodelon	876	NA	NA	NA	1100	NA	NA	NA	787	965	1036	1164	0.81
Jin	420	208	160	52	842	543	257	42	576	264	1343	341	0.111
Lai	120	98	14	8	100	97	2	1	210	30	196	4	**<0.005**
Pandey	200	59	115	26	200	59	112	29	233	167	230	170	**0.039**
Roszak	426	87	230	109	460	122	235	103	404	448	479	441	0.614
XU	345	135	163	47	330	131	152	47	433	257	414	246	0.786
Zidi	130	42	48	40	260	83	117	60	132	128	283	237	0.134

−1486T>C	Total	TT	TC	CC	Total	TT	TC	CC	T	C	T	C	HWE
Bi	102	25	68	9	100	26	54	20	118	86	106	94	0.401
Chen	694	246	346	102	715	289	319	107	838	550	897	533	0.220
Lai	120	118	1	1	100	99	0	1	237	3	198	2	**<0.005**
Roszak	426	141	206	79	460	193	203	64	488	364	589	331	0.367

HWE: Hardy-Weinberg equilibrium.

**Table 3:** Meta-analysis results of rs352140 based on five genetic models.

Genetic models	OR (95% CI)	*p* value	Heterogeneity		Effects model
			*I * ^2^ (%)	*p* value	
Allele model (A versus G)					
Overall	1.20 (0.97, 1.47)	0.09	83	0.00001	R
Race					
Chinese Han	1.46 (0.86, 2.48)	0.16	91	<0.0001	R
Caucasian	1.11 (1.01, 1.22)	**0.03**	**0**	0.62	R

Dominant model (AA + GA versus GG)					
Overall	1.30 (0.97, 1.74)	0.08	73	0.001	R
Race					
Chinese Han	1.54 (0.91, 2.61)	0.11	83	0.0006	R
Caucasian	1.17 (0.91, 1.49)	0.22	**17**	0.30	R

Recessive model (AA versus GA + GG)					
Overall	1.23 (0.81, 1.86)	0.34	77	0.0002	R
Race					
Chinese Han	1.35 (0.54, 3.39)	0.52	87	<0.0001	R
Caucasian	1.20 (0.94, 1.51)	0.14	**0**	0.39	R

Heterozygous genetic model (GA versus GG)					
Overall	1.24 (0.96, 1.59)	0.10	58	0.03	R
Race					
Chinese Han	1.42 (0.94, 2.15)	0.09	67	0.03	R
Caucasian	1.09 (0.81, 1.49)	0.56	**38**	0.20	R

Homozygous genetic model (AA versus GG)					
Overall	1.34 (0.83, 2.15)	0.23	78	0.0001	R
Race					
Chinese Han	1.53 (0.57, 4.15)	0.40	88	<0.0001	R
Caucasian	1.30 (0.98, 1.73)	0.07	**0**	0.42	R

F: fixed-effect model; R: random-effect model; OR: odds ratio; 95% CI: 95% confidence interval.

**Table 4:** Meta-analysis results of rs187084 based on five genetic models.

Genetic models	OR (95% CI)	*p* value	Heterogeneity		Effects model
			*I * ^2^ (%)	*p* value	
Allele model (C versus T)					
Overall	1.15 (1.03, 1.29)	**0.02**	43	0.15	F
Race					
Chinese Han	1.06 (0.92, 1.23)	0.39	0	0.38	F
Caucasian	1.33 (1.10, 1.61)	0.004	—	—	—

Dominant model (CC + CT versus TT)					
Overall	1.30 (1.11, 1.53)	**0.002**	0	0.73	F
Race					
Chinese Han	1.22 (1.00, 1.50)	**0.05**	0	0.90	F
Caucasian	1.46 (1.11, 1.92)	**0.007**	—	—	—

Recessive model (CC versus CT + TT)					
Overall	0.94 (0.59, 1.50)	0.79	63	0.04	R
Race					
Chinese Han	0.70 (0.34, 1.44)	0.34	52	0.12	R
Caucasian	1.41 (0.98, 2.02)	0.06	—	—	—

Heterozygous genetic model (CT versus TT)					
Overall	1.32 (1.11, 1.57)	**0.002**	0	0.95	F
Race					
Chinese Han	1.28 (1.03, 1.59)	**0.02**	0	0.92	F
Caucasian	1.39 (1.04, 1.86)	**0.03**	—	—	—

Homozygous genetic model (CC versus TT)					
Overall	1.13 (0.71, 1.80)	0.60	56	0.08	R
Race					
Chinese Han	0.88 (0.49, 1.59)	0.67	**31**	0.24	R
Caucasian	1.69 (1.14, 2.51)	0.009	—	—	—

F: fixed-effect model; R: random-effect model; OR: odds ratio; 95% CI: 95% confidence interval.

## Data Availability

The data used to support the findings of this study are available from the corresponding author upon request.
